# P-2325. Can activated partial thromboplastin time (aPTT) be used as a marker to predict bleeding and severity of Dengue fever in resource limited setting?

**DOI:** 10.1093/ofid/ofae631.2477

**Published:** 2025-01-29

**Authors:** Ashish Bhalla, Ashish Kumar Bhalla, Ashok Kumar, Vikas Suri, Harpreet Singh, Debaprasad Dibhar, Navneet Sharma

**Affiliations:** PGIMER, Chandigarh, Chandigarh, India; Post Graduate Institute of medical education and Research, Chandigarh, Chandigarh, India; Post Graduate Institute of medical education and Research, Chandigarh, Chandigarh, India; PGIMER, CHANDIGARH, INDIA, Chandigarh, Chandigarh, India; PGIMER, Chandigarh, Chandigarh, India; Post Graduate Institute of medical education and Research, Chandigarh, Chandigarh, India; Post Graduate Institute of medical education and Research, Chandigarh, Chandigarh, India

## Abstract

**Background:**

Bleeding in Dengue has been attributed to dysfunction in intrinsic pathway, however, there are no clear evidence of its use as a predictor of severity or mortality in adult patients with Dengue fever.

Our hypothesis is that prolonged aPTT is associated with life threatening bleeding and severity of Dengue fever.

**Methods:**

a retrospective analysis of all patients diagnosed as Dengue fever admitted during the months of July to October 2023 was undertaken. The diagnosis of Dengue fever was established based on NS1 antigen positive (day 1-5) and/or IgM positive (day 7-12). Patients with dual infections, alternate diagnosis or secondary Dengue (IgG positive) were excluded. Details of the clinical parameters, haemogram, coagulation factors, liver and kidney function tests were recorded. The patients were classified as dengue without warning signs (mild), Dengue with warning signs (moderate) and dengue with Dengue shock syndrome(DSS)/ (DHF)Dengue hemorrhagic fever (severe) on the day of presentation. Progression to DSS/DHF during hospital stay, life threatening bleeding and mortality were the outcomes studied.

**Results:**

195 patients were admitted but only 163 with complete clinical detail/ laboratory investigations were included in the study. The mean age was 31.8 years (range 13-80 years). Male : female ratio was 56%:44%. Average interval between onset of symptoms to clinical presentation was 7 days (range 5-11 days). Average duration of stay was 6.8 days. 80 patients (49%) had severe dengue at the time of presentation. 71 patients (43%) had bleeding with less than of them 50% having clinically significant bleeding. 103 patients (63%) patients had prolonged aPTT ( >35 seconds) at admission. 52 patients (32%) died. On multivariate analysis, prolonged aPTT and a low platelet count (< 10,000) at admission was not found to be associated with either clinically significant bleeding, severity of illness or mortality.

**Conclusion:**

Severity, clinically significant bleeding and mortality in Dengue is not associated with either platelet count or APTT at presentation.

Limitation: our study included parameters at admission and did not include the lowest platelet counts and highest values of aPTT during admission.

**Disclosures:**

All Authors: No reported disclosures

